# Low skeletal muscle mass is associated with inferior preoperative and postoperative shoulder function in elderly rotator cuff tear patients

**DOI:** 10.1186/s12877-024-05209-5

**Published:** 2024-07-20

**Authors:** Yang Yang, Binbin Zheng, Xiaofang Lin, Mengqin Zhang, Yongzhi Ye, Haixiao Chen, Xiaobo Zhou

**Affiliations:** 1 Department of Orthopedics, Taizhou Hospital of Zhejiang Province, Affiliated to Wenzhou Medical University, No. 150 Ximen Street, Linhai City, 317000 Zhejiang Province China; 2 Department of Critical Care Medicine, Taizhou Hospital of Zhejiang Province, Affiliated to Wenzhou Medical University, No. 150 Ximen Street, Linhai City, 317000 Zhejiang Province China

**Keywords:** Rotator cuff tear, Sarcopenia, Elderly patients, Shoulder function, Skeletal muscle mass

## Abstract

**Background:**

The age-related loss of skeletal muscle mass is an important characteristic of sarcopenia, an increasingly recognized condition with systemic implications. However, its association with shoulder function in elderly patients with rotator cuff tears (RCT) remains unknown. This study aimed to investigate the relationship between low skeletal muscle mass and shoulder function in elderly RCT patients.

**Methods:**

A retrospective analysis was conducted on RCT patients who underwent chest computed tomography (CT) scans for clinical evaluation. Preoperative CT scan images of the chest were used to calculate the cross-sectional area (CSA) of thoracic muscle at the T4 level. The medical records were reviewed. Shoulder function was assessed using the ASES score and CMS score both preoperatively and at the final follow-up. Data on the preoperative range of motion (ROM) for the affected shoulder, were collected for analysis. Subgroup analyses by sex were also performed.

**Results:**

A total of 283 RCT patients, consisting of 95 males and 188 females, with a mean age of 66.22 ± 4.89(range, 60–95 years) years were included in this retrospective study. The low muscle mass group showed significantly higher level of c-reactive protein (CRP) and erythrocyte sedimentation rate (ESR) compared to the normal group(3.75 ± 6.64 mg/L vs. 2.17 ± 2.30 mg/L, *p* = 0.021; 19.08 ± 12.86 mm/H vs.15.95 ± 10.76 mm/H, *p* = 0.038; respectively). In the normal group, pre-operative passive ROM, including forward elevation, abduction, lateral rotation, and abductive external rotation, was significantly better than that in the low muscle mass group (127.18 ± 34.87° vs. 89.76 ± 50.61°; 119.83 ± 45.76° vs. 87.16 ± 53.32°; 37.96 ± 28.33° vs. 25.82 ± 27.82°; 47.71 ± 23.56° vs. 30.87 ± 27.76°, all *p* < 0.01, respectively). Similar results were found in the active ROM of the shoulder. The female low muscle mass group exhibited significantly poorer passive and active ROM (*p* < 0.05). The post-operative ASES scores and CMS scores of the female low muscle mass group were also statistically worse than those of the female normal group (*p* < 0.05).

**Conclusions:**

The results of present study revealed that the low skeletal muscle mass is associated with inferior ROM of the shoulder and per- and post-operative shoulder function, especially for elderly female patients.

## Background

Sarcopenia, characterized by the age-related progressive decreases in skeletal muscle mass, strength and muscle function, can lead to adverse outcomes such as physical disability, poor quality of life, and death [1–3]. Sarcopenia is initially proposed by Rosenberg et al. in 1989 [4]. Over the past two decades, it has gained widespread recognition as a distinct geriatric syndrome. The understanding of sarcopenia, including its causes, underlying mechanisms, and risk factors, has significantly expanded [1, 5, 6].

It was reported that sarcopenia is strongly associated with systemic diseases such as cardiovascular disease, obesity, rheumatoid arthritis, and osteoporosis [5, 7–9]. While the rotator cuff tear (RCT) is a localized condition affecting tendons and skeletal muscles in the shoulder. A chronic RCT may result in shoulder dysfunction and atrophy of the skeletal muscles around the shoulder. The relationship between sarcopenia and RCT has been investigated in studies conducted by Chung et al. [10], Atala et al. [11] and Han et al. [12]. But no consistent conclusion has been reached on this matter. Chung et al. conducted a study including 48 chronic symptomatic full-thickness RCT patients, with a mean age of 60.1 ± 6.5 years, range from 46 to 76 years, and compared them with 48 age- and sex-matched control patients [10]. They discovered that sarcopenic index was significantly lower in RCT patients, and the large to massive RCT patients have lower sarcopenic index than small to medium RCT patients. On the other hand, Atala et al. performed a prospective case-control study with 53 consecutive RCT patients, all aged 65 years or older, with a mean age of 72 ± 5 years, and compared them with 53 age- and sex-matched non-RCT patients [11]. Their conclusion was that there was no relationship between prevalence of sarcopenia and RCT. The age-related loss of skeletal muscle mass is an important characteristic of sarcopenia. The question of whether low skeletal muscle mass is associated with RCT, particularly in cases of large to massive RCT, and whether RCT patients with concomitant low skeletal muscle mass experience clinical outcomes as satisfactory as RCT patients with normal skeletal muscle mass remains unknown.

The diagnosis of sarcopenia requires measurements of skeletal muscle quantity, muscle strength and physical performance [2, 13, 14]. Numerous methods have been reported to assess the body skeletal muscle mass, including multifrequency bioelectrical impedance analysis (BIA), dual-energy X-ray absorptiometry (DXA), magnetic resonance imaging (MRI) and computed tomography (CT) [2, 13]. DXA, MRI and CT have been recognized with high precision for muscle mass measurement. Traditionally, the third level of lumbar of a CT scan was employed to assess body muscle mass, but it is not routinely performed for RCT patients. Instead, the level 4 thoracic cross-sectional area (T4CSA) for muscle measurement has been used for sarcopenia and thoracic skeletal muscles study. Furthermore, Moon et al. conducted a retrospective study involving 4470 Asian patients to establish cutoff values for T4CSA and the level 4 thoracic muscle index (T4MI) in the diagnosis of sarcopenia [15]. Their findings revealed that for males, the T4CSA cutoff value was 100.06 cm², and for females, it was 66.93 cm². Additionally, the T4MI cutoff values were 33.69 cm²/m² for males and 26.01 cm²/m² for females [15]. Since chest CT scans were routinely performed for RCT patients at our institution, this approach proved convenient for both patients and clinicians in assessing sarcopenia. In present study, we conducted a retrospective review of chest CT scans from RCT patients at our institution to investigate the relationship between the low skeletal muscle mass and the pre- and post-operative shoulder function of elderly RCT patients.

## Method

In this retrospective study, we reviewed patients who underwent the arthroscopy assisted rotator cuff repair because of RCT from 1st February 2021 to 31th January 2022. RCT was initially diagnosed using preoperative MRI and subsequently confirmed during surgery. The tear size of the rotator cuff was measured intraoperatively and classified into four categories: small tears (< 1 cm), medium tears (> 1 to < 3 cm), large tears (> 3 to < 5 cm), and massive tears (> 5 cm). All patients underwent medial single-row rotator cuff repair. Depending on the patient’s shoulder stiffness, we performed shoulder joint release, including the rotator cuff interval, the middle glenohumeral ligament, and a 270-degree release of the shoulder joint capsule.

Inclusion criteria: (1) Age ≥ 60years; (2) Patients with RCT who underwent arthroscopy-assisted surgery; (3) Availability of preoperative chest CT scans for review; (4) Follow-up period of ≥ 12months; (5) The CT scan image data were restored in authors’ institution. Exclusion criteria: (1) Lack of preoperative chest CT scans; (2) Absence of preoperative or postoperative functional assessments; (3) Lost to follow-up.

**Fig. 1:**
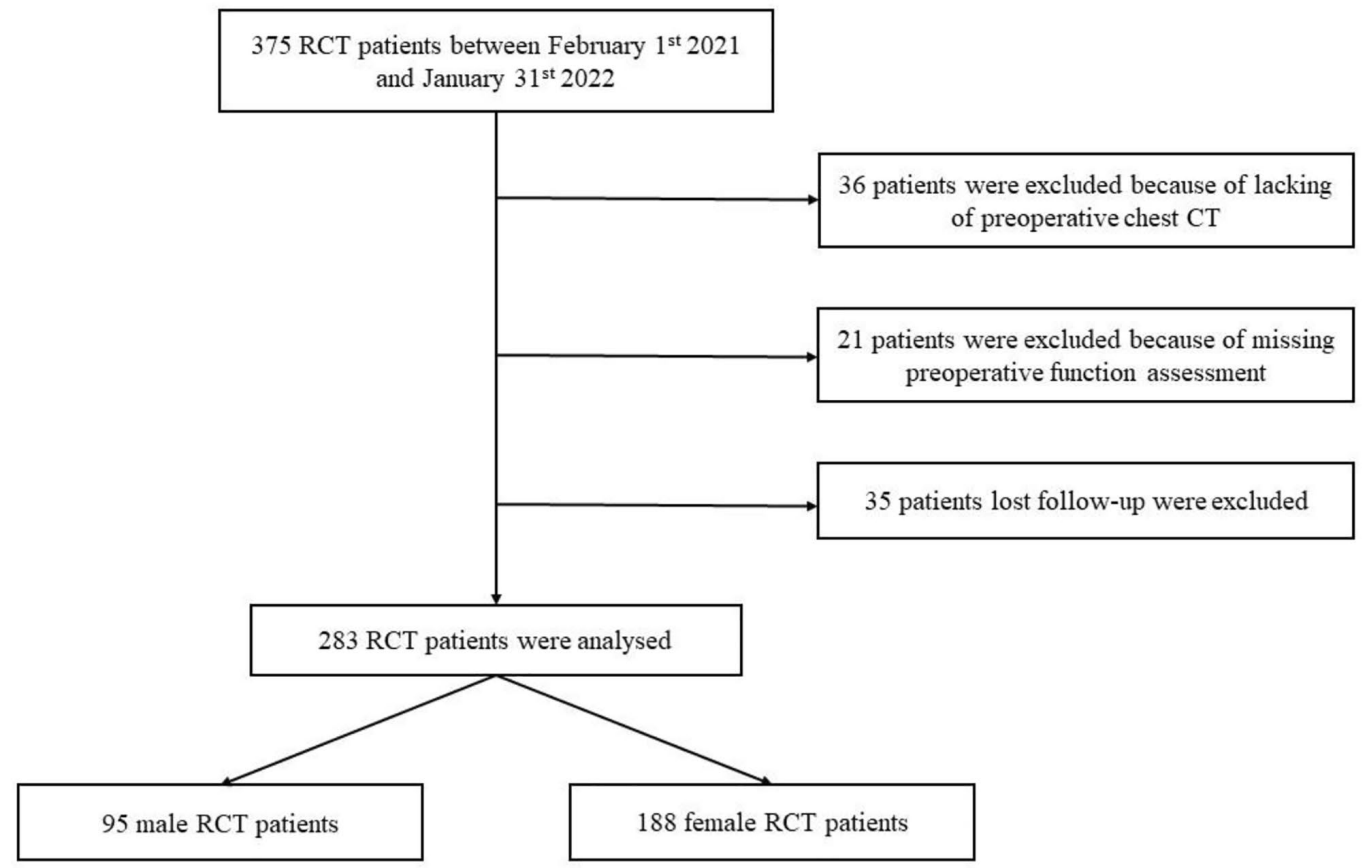
Flow diagram of rotator cuff tear patients in present study. Abbreviations: RCT, rotator cuff tear; CT, computed tomography

A flow diagram illustrating the participant selection process for this study is presented in Fig. 1. Initially, 375 RCT patients aged 60 and above were reviewed. Subsequently, 36 patients were excluded due to a lack of preoperative chest CT scans, 21 patients were excluded because of inadequate preoperative functional assessment; and 35 patients were excluded for missing follow up. Ultimately, a total of 283 RCT patients, consisting of 95 males and 188 females, were analyzed in this study.

The medical records of these patients were meticulously reviewed by two independent authors. Information on comorbidities such as hypertension and diabetes, the mechanism of injury, operative side, body mass index (BMI), and preoperative hematological examination including haemoglobin, white blood cell, C-reactive protein (CRP), erythrocyte sedimentation rate (ESR), creatinine, alanine aminotransferase, albumin were collected for further analysis. Shoulder function was assessed using the ASES score and CMS score both preoperatively and at the final follow-up. Additionally, data on the preoperative range of motion (ROM) for the affected shoulder, including active ROM and passive ROM, were collected for analysis.

**Fig. 2:**
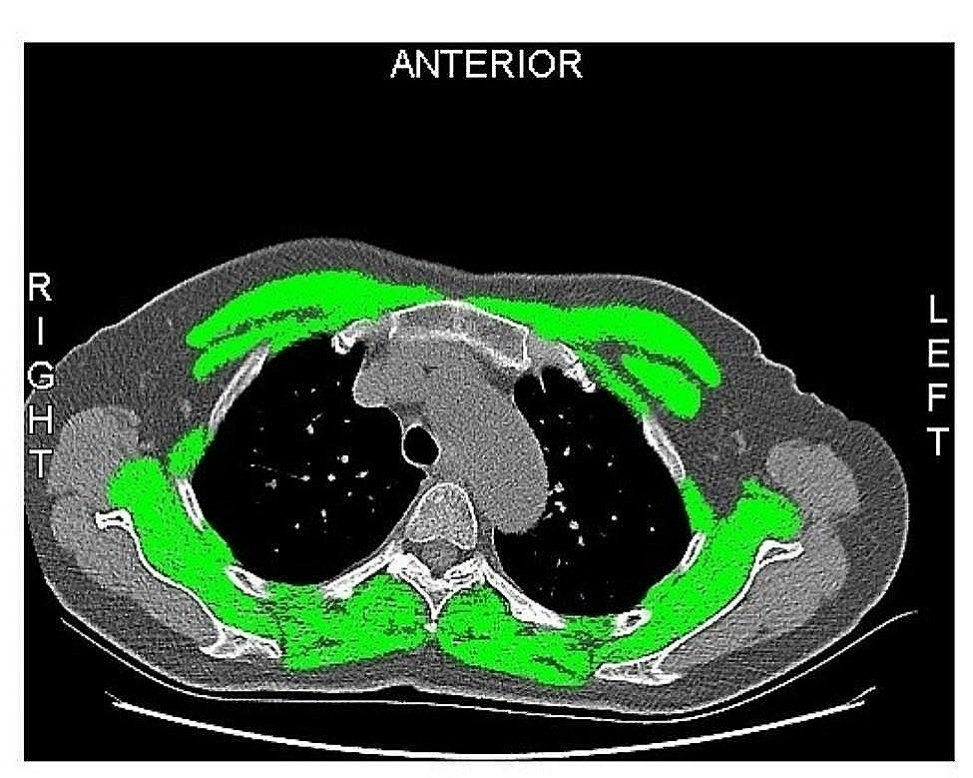
Illustrating the measurement of muscle at the 4th thoracic level cross-sectional. The green region is the total cross-sectional area of pectoralis, intercostalis, paraspinals, serratus, and latissimus muscles

Preoperative CT scan images of the chest were used to calculate the T4CSA. The thoracic muscle of T4 level encompassed the pectoralis, intercostalis, paraspinal, serratus, and latissimus muscles [16]. To ensure consistency, all chest CT scans followed standardized positioning guidelines, with the arms aligned alongside the trunk, adhering to the Health Promotion Center of Severance Hospital’s protocol. The T4 level slice is characterized by an ascending aorta and a reversed heart-shaped appearance of the T4 vertebra (Fig. 2). The CT slice at the T4 level was confirmed and exported by a senior physician. These CT scan images were exported in DICOM format and subsequently imported into sliceOmatic 5.0 (TomoVision, Canada) for a semi-automated quantitative assessment (Fig. 2). The pixel attenuation of skeletal muscle was set within the range of -30 to 150 Hounsfield units. The T4CSA of skeletal muscle was standardized via dividing the T4CSA (cm^2^) by patients’ height squared (m^2^) and this standardized data was defined as T4 level skeletal muscle index (T4MI, cm^2^/m^2^) [16]. Since this study was designed retrospectively, we could not have access to measurements such as handgrip strength, gait speed, BIA or DXA to measure sarcopenia. Therefore, we used the reference values of skeletal muscle at T4 level to determine the sarcopenia. In present study, male patients with a T4MI less than 33.69cm^2^/m^2^ and female patients with a T4MI less than 26.01cm^2^/m^2^ were classified into low muscle mass group.

### Statistical analysis

The statistical analysis was performed using SPSS version 26.0 (SPSS Inc., Chicago, IL, USA). Descriptive statistics were presented as either numbers with proportions or as means with standard deviations (SDs). Chi-square tests or Fisher’s exact test were conducted to compare categorical variables; students’ *t*-tests were performed to compare continuous variables between the two groups. A *p*-value < 0.05 was regarded as statistically significant.

## Results

### Baseline characteristics

A total of 283 RCT patients, consisting of 95 males and 188 females, with a mean age of 66.22 ± 4.89 (range, 60–95 years) years were included in this retrospective study. The baseline data, including age, BMI, operation side, hypertension, diabetes, CRP, ESR, creatinine, alanine aminotransferase, albumin, the number of massive RCT, injury mechanism, and follow-up period, showed no significant differences between the male and female groups. No significant difference was found between males and females in regarding of T4CAS, T4MI and the prevalence of sarcopenia. However, the male group exhibited statistically higher levels of hemoglobin and creatinine than the female group (*p* < 0.01). Conversely, the ESR of male group was statistically lower than female group (Table 1).

**Table 1:** Patient characteristics

Variable	Total	Male	Female	*P*-value
Number of patients(n)	283	95	188	
Age(years)	66.22 ± 4.89	66.33 ± 5.93	66.16 ± 4.28	0.878
BMI(kg/m^2^)	24.34 ± 3.88	23.79 ± 2.48	24.62 ± 4.40	0.089
Operative side(left/right)	77/206	49/139	28/67	0.543
Hypertension(n)	84	32	52	0.295
Diabetes(n)	42	12	30	0.457
Haemoglobin(g/L)	142.10 ± 13.51	152.22 ± 13.27	136.98 ± 10.41	< 0.001
White blood cell(10^9^/L)	6.42 ± 1.74	6.51 ± 1.90	6.37 ± 1.66	0.531
CRP(mg/L)	2.75 ± 4.47	2.07 ± 2.33	3.10 ± 5.20	0.067
ESR (mm/H)	17.10 ± 11.65	11.31 ± 8.01	20.03 ± 12.12	< 0.001
Creatinine(umol/L)	63.54 ± 12.67	73.84 ± 10.42	58.34 ± 10.32	< 0.001
Alanine aminotransferase(U/L)	22.82 ± 13.76	23.09 ± 10.37	22.69 ± 15.20	0.814
Albumin(g/L)	45.31 ± 3.98	45.38 ± 2.63	45.28 ± 4.51	0.839
Massive rotator cuff tear(n)	98	68	30	0.443
Injury(n)	121	41	80	0.923
Follow-up time(mon)	17.12 ± 3.78	16.88 ± 3.85	17.59 ± 3.63	0.135
T4CSA (cm^2^)	90.97 ± 20.71	108.54 ± 19.94	82.09 ± 14.52	< 0.001
T4MI (cm^2^/m^2^)	30.81 ± 10.53	35.54 ± 11.13	28.43 ± 9.38	< 0.001
Low muscle mass (n)	104	33	71	0.618

BMI: Body mass index; CRP: C-reactive protein; ESR: Erythrocyte sedimentation rate; T4CSA: Level 4 thoracic cross-sectional area; T4MI: T4 muscle index = T4CSA/height^2^

### General data comparisons

According to the T4MI cutoff value for diagnosis of sarcopenia, all patients were divided into either the normal group or the low muscle mass group. The normal group consisted of 179 patients and the low muscle mass group had 104 patients. No significant differences were observed in terms of age, sex, BMI, operative side, hypertension, diabetes, hemoglobin, white blood cell count, creatinine, alanine aminotransferase, albumin, the number of massive RCT, and injury mechanism between these two groups. However, the low muscle mass group showed significantly higher level of CRP and ESR compared to the normal group. (3.75 ± 6.64 mg/L vs. 2.17 ± 2.30 mg/L, *p* = 0.021; 19.08 ± 12.86 mm/H vs.15.95 ± 10.76 mm/H, *p* = 0.038; respectively). A total of 196 patients did not undergo joint release, while 87 patients did, including 35 in the low muscle mass group and 52 in the normal group. There was no significant difference between two groups (*p* = 0.418) (Table 2).

**Table 2:** General data comparisons between low muscle mass and normal groups

Variable	Normal *(n* = 179)	Low muscle mass (*n* = 104)	*P*-value
Age(years)	66.18 ± 5.04	66.27 ± 4.64	0.888
Sex(male/female)	62/117	33/71	
BMI (kg/m^2^)	24.45 ± 2.93	24.15 ± 5.13	0.594
Operative side(left/right)	53/126	24/80	0.234
Hypertension(n)	55	29	0.614
Diabetes(n)	31	11	0.124
Haemoglobin(g/L)	142.27 ± 12.81	141.79 ± 14.69	0.771
White blood cell(10^9^/L)	6.34 ± 1.73	6.54 ± 1.77	0.360
CRP(mg/L)	2.17 ± 2.30	3.75 ± 6.64	0.021
ESR(mm/H)	15.95 ± 10.76	19.08 ± 12.86	0.038
Creatinine(umol/L)	63.98 ± 13.17	62.79 ± 11.79	0.445
Alanine aminotransferase(U/L)	22.41 ± 13.89	23.54 ± 13.55	0.506
Albumin(g/L)	25.29 ± 4.50	45.36 ± 2.88	0.875
Massive rotator cuff tear(n)	57/122	41/63	0.196
Injury(n)	69/110	52/52	0.060
Joint release(n)	52	35	0.418

BMI: Body mass index; CRP: C-reactive protein; ESR: Erythrocyte sedimentation rate;

### Shoulder functional comparisons

We conducted a comparison of both pre-operative passive and active ROM between the normal group and the low muscle mass group. To assess shoulder function, we used pre-operative and post-operative ASES scores and CMS scores. In the normal group, pre-operative passive ROM, including forward elevation, abduction, lateral rotation, and abductive external rotation, was significantly greater than that in the low muscle mass group (127.18 ± 34.87° vs. 89.76 ± 50.61°, *p* < 0.01; 119.83 ± 45.76° vs. 87.16 ± 53.32°, *p* < 0.01; 37.96 ± 28.33° vs. 25.82 ± 27.82°, *p* < 0.01; 47.71 ± 23.56° vs. 30.87 ± 27.76°, *p* < 0.01, respectively). Similar results were found in the active ROM of the shoulder, where forward elevation, abduction, lateral rotation, abductive external rotation, and adduction in the normal group were significantly better than in the low muscle mass group (145.47 ± 26.25° vs. 118.75 ± 37.05°; 137.63 ± 38.25° vs. 110.58 ± 46.70°; 44.25 ± 29.29° vs. 31.01 ± 28.69°; 56.40 ± 24.88° vs. 39.23 ± 30.44°; 30.14 ± 5.73 mm vs. 32.94 ± 6.63 mm; respectively, all *p* < 0.01). Moreover, pre-operative ASES score and post-operative CMS score in normal group were significantly better than those in the low muscle mass group. Interestingly, both the post-operative ASES score and CMS score of the normal group were significantly superior to those in the low muscle mass group (89.99 ± 6.14 vs. 87.20 ± 8.89, *p* = 0.002; 88.46 ± 7.30 vs. 85.99 ± 8.51, *p* = 0.01; respectively) (Table 3).

**Table 3:** Pre-and post-operative functional comparisons between low muscle mass and normal groups

Variable	Normal	Low muscle mass	*P*-value
Pre-op. passive range of motion			
Forward elevation(°)	127.18 ± 34.87	89.76 ± 50.61	<0.001
Abduction(°)	119.83 ± 45.76	87.16 ± 53.32	<0.001
Lateral rotation(°)	37.96 ± 28.33	25.82 ± 27.82	0.001
Abductive external rotation(°)	47.71 ± 23.56	30.87 ± 27.76	<0.001
Pre-op. active range of motion			
Forward elevation(°)	145.47 ± 26.25	118.75 ± 37.05	<0.001
Abduction(°)	137.63 ± 38.25	110.58 ± 46.70	<0.001
Lateral rotation(°)	44.25 ± 29.29	31.01 ± 28.69	<0.001
Abductive external rotation(°)	56.40 ± 24.88	39.23 ± 30.44	<0.001
Adduction(mm)	30.14 ± 5.73	32.94 ± 6.63	<0.001
Pre-op. ASES score	35.29 ± 13.33	27.87 ± 14.81	<0.001
Pre-op. CMS score	55.60 ± 14.37	39.23 ± 19.11	<0.001
Post-op. ASES score	89.99 ± 6.14	87.20 ± 8.89	0.002
Post-op. CMS score	88.46 ± 7.30	85.99 ± 8.51	0.01

Pre-op.: preoperative; Post-op.: postoperative

To mitigate the influence of sex-related differences in sarcopenia, we conducted separate analyses of shoulder function for male and female patients. The results revealed that male patients in the normal group exhibited significantly better passive and active forward elevation, as well as both pre-operative and post-operative abductive external rotation, compared to the low muscle mass group (*p* < 0.05). Additionally, male patients in the normal group displayed significantly better adduction than those in the male sarcopenia group. However, no statistically significant differences were observed between the male normal group and male low muscle mass group regarding both passive and active abduction and lateral rotation (*p* > 0.05). Notably, although the pre-operative CMS scores of male patients in the low muscle mass group was statistically worse than that of the male normal group, both post-operative ASES scores and CMS scores for male patients in the low muscle mass group were similar to those of male patients in the normal group (88.82 ± 6.52 vs. 90.95 ± 4.80, *p* = 0.072; 87.21 ± 7.29 vs. 89.21 ± 6.10, *p* = 0.159, respectively) (Table 4).

**Table 4:** Pre-and post-operative subgroup functional comparisons between low muscle mass and normal groups

Variable		Male				Female	
	Normal (*n* = 62)	Low muscle mass (*n* = 33)	*P*-value		Normal (*n* = 117)	Low muscle mass (*n* = 71)	*P*-value
**Pre-op. passive range of motion**							
Forward elevation(°)	145.40 ± 24.38	119.85 ± 32.51	<0.001		145.51 ± 27.30	118.24 ± 39.19	<0.001
Abduction(°)	132.10 ± 40.17	113.79 ± 45.26	0.046		140.56 ± 37.03	109.08 ± 47.60	<0.001
Adductive external rotation(°)	43.79 ± 27.34	39.39 ± 23.07	0.434		44.487 ± 30.38	27.117 ± 30.32	<0.001
Abductive external rotation(°)	56.45 ± 24.17	44.55 ± 25.35	0.027		56.37 ± 25.35	36.76 ± 32.41	<0.001
**Pre-op. active range of motion**							
Forward elevation(°)	126.29 ± 37.54	95.91 ± 45.32	0.001		127.65 ± 33.53	86.90 ± 52.95	<0.001
Abduction(°)	110.81 ± 45.46	92.12 ± 50.36	0.069		124.62 ± 45.38	84.86 ± 54.84	<0.001
Adductive external rotation(°)	39.44 ± 27.10	35.15 ± 24.42	0.450		37.18 ± 29.05	21.48 ± 28.39	<0.001
Abductive external rotation(°)	49.52 ± 22.52	35.30 ± 22.29	0.004		46.75 ± 24.13	28.80 ± 29.89	<0.001
Adduction(mm)	31.56 ± 5.66	34.85 ± 6.57	0.018		29.38 ± 5.64	32.06 ± 6.52	0.03
Pre-op. ASES score	35.54 ± 11.94	33.74 ± 17.49	0.599		35.158 ± 14.06	25.14 ± 12.61	<0.001
Pre-op. CMS score	54.65 ± 13.92	44.09 ± 19.42	0.008		56.11 ± 14.64	36.97 ± 18.67	<0.001
Post-op. ASES score	90.95 ± 4.80	88.82 ± 6.52	0.072		89.49 ± 6.71	86.45 ± 9.75	0.012
Post-op. CMS score	89.21 ± 6.10	87.21 ± 7.29	0.159		88.07 ± 7.86	85.42 ± 9.01	0.036

Pre-op.: preoperative; Post-op.: postoperative

In contrast to male patients, female patients appeared to be more susceptible to sarcopenia. The female low muscle mass group showed significantly poorer passive and active ROM (*p* < 0.05). Additionally, pre-operative shoulder functional assessments, including ASES scores and CMS scores, were significantly lower in the female low muscle mass group compared to the female normal group (*p* < 0.05). Furthermore, the post-operative ASES scores and CMS scores of the female low muscle mass group were also significantly worse than those of the female normal group (*p* < 0.05) (Table 4).

## Discussion

In this retrospective study, we sought to investigate the connection between sarcopenia and shoulder function in patients with RCTs. Our choice to employ T4MI as a reference for sarcopenia determination was informed by the routine use of chest CT scans in clinical practice, their ready availability, and their established utility in measuring muscle mass, which aids in sarcopenia diagnosis. Factors previously identified as influencing shoulder function in RCT patients encompass age, RCT size, chronic pain, kinesiophobia, anxiety, and depression [17, 18]. Nevertheless, the connection between sarcopenia and shoulder function in elderly RCT patients has not been thoroughly studied in existing literature. To the best of our knowledge, this is the first study using T4MI reference for sarcopenia diagnosis to investigate the association between sarcopenia and shoulder function of elderly RCT patients. Our findings revealed that elderly RCT patients with sarcopenia exhibited higher levels of CRP (3.75 ± 6.64 mg/L vs. 2.17 ± 2.30 mg/L, *p* = 0.021) and ESR (19.08 ± 12.86 mm/H vs. 15.95 ± 10.76 mm/H, *p* = 0.038) than those in the normal group. When comparing shoulder function, both passive and active ranges of motion were significantly inferior in the low muscle mass group compared to the normal RCT patient group. Additionally, ASES scores and CMS scores were more favorable in the normal group following the operation.

Chronic inflammation is widely acknowledged as a fundamental pathological condition that contributes to the initiation and advancement of various chronic illnesses [19–22]. Moreover, skeletal muscle tissue dysfunction can also trigger a chronic inflammatory response, subsequently impairing skeletal muscle function, leading to a detrimental cycle [23]. Previous studies have indicated that elevated levels of inflammatory markers in the blood would exacerbate the loss of skeletal muscle mass and strength, accompanied with lower physical activity [24, 25]. Researchers found that muscle degeneration was extensive, with 90% of muscle bundles showing signs of damage, and 74% of biopsy samples containing no muscle tissue at all, despite targeting muscle during surgery. They suggested that imbalanced muscle degeneration-regeneration, potentially worsened by inflammation, is a primary cause of muscle loss in late-stage rotator cuff disease. This muscle loss can result in reduced skeletal muscle strength [26, 27]. Additionally, researches have shown a correlation between the reduction in skeletal muscle mass, strength, and limb-related physical fitness in both genders and elevated levels of proinflammatory cytokines, including IL-6, tumor necrosis factor-α, and CRP [24, 28]. Currently, there is substantial evidence suggesting that age-related low-grade chronic inflammation may play a pivotal role in the development of sarcopenia [29]. Even this study found that CRP and ESR levels were significantly higher in the low muscle mass group compared to the normal group. Present evidence makes it challenging to definitively conclude whether patients with low skeletal muscle mass experience chronically elevated inflammatory markers.

In present study, we found that RCT patients with sarcopenia showed lower pre-operative ASES scores and CMS scores and inferior post-operative ASES scores and CMS scores. Additionally, sarcopenia was associated with poorer passive and active ROM of the shoulder. Chung et al. reported that sarcopenia is more severe in patients with chronic symptomatic full-thickness RCT compared to age- and sex-matched control populations [10]. Moreover, their research indicated that the severity of sarcopenia was correlated with the size of the RCT. It’s worth noting that Chung et al.’s study included participants under the age of 65, which differs from the recommendation of the European Working Group and Sarcopenia in Older People (EWGSOP2), which suggests a cutoff age of 65 years or older for primary sarcopenia diagnosis [1]. Sarcopenia can also be found in young adulthood and is commonly classified as secondary sarcopenia, attributed to factors such as metabolic syndrome, inadequate nutrition, vitamin D deficiency, endocrine disorders, among others [30]. This difference in age inclusion criteria may have implications for the generalizability of their findings to older populations, particularly given the age-related nature of sarcopenia.

In contrast to the findings of Chung et al., the study performed by Atala et al. did not find a significant correlation between RCT and sarcopenia. Their research revealed that factors like gait speed, grip strength, and skeletal muscle mass index did not exhibit significant differences between patients with RCT and those without. Additionally, the size of the RCT did not significantly affect these measures. These contrasting results between the two studies underline the need for further investigation to gain a comprehensive understanding of the relationship between sarcopenia and RCT, considering various factors and age groups. This discrepancy in findings may be attributed to differences in the study populations, possibly influenced by varying ethnic backgrounds. Moreover, both Chuang et al. and Atala et al. investigated the difference prevalence of sarcopenia between full thick RCT patients and those without and try to descript the correlation of sarcopenia and RCT. It’s essential to highlight this logical relationship in the context of previous research. In present study, we divided all these RCT patients into two groups basing on sarcopenia or not. Interestingly, we did not find the significant correlation between sarcopenia and the size of RCT.

To minimize the influence of gender, we analyzed male and female patients separately (as shown in Table 4). The results showed that male RCT patients with sarcopenia had a similar satisfactory ASES scores and CMS scores after surgery compared to patients without sarcopenia. However, the post-operative ASES scores (86.45 ± 9.75 vs. 89.49 ± 6.71, *p* = 0.012) and CMS scores (85.42 ± 9.01 vs. 88.07 ± 7.86, *p* = 0.036) of female RCT patients with sarcopenia were significantly inferior to female RCT patients without sarcopenia. This founding indicates that sarcopenia may have a more pronounced impact on the clinical outcomes of female RCT patients. Numerous literatures indicated that females are more susceptible to kinesiophobia and tend to experience poorer clinical functional recovery compared to males [31–33]. Studies have identified female sex as a significant independent risk factor for rotator cuff tears [34, 35]. The generally lax shoulder joints in females may contribute to the development of rotator cuff tendinopathy. Additionally, genetic variations in females may alter the extracellular matrix structure of rotator cuff tissues, increasing their susceptibility to RCT. Figueiredo et al. reported that females are more frequently affected by the T allele of MMP2, the T allele of MMP3, and the TT genotype of TIMP2, which are associated with RCT [36]. Furthermore, females with RCT often report more severe clinical symptoms than males [37]. Following rotator cuff repair surgery, females are more prone to experiencing shoulder pain and slower functional recovery, which may be associated with sex hormone levels [38, 39]. Therefore, it is crucial for clinicians to place particular emphasis on the rehabilitation of RCT patients with sarcopenia, especially for female patients, to ensure optimal recovery and functional outcomes.

Several limitations of this study should be noted before interpreting the results. First, the diagnostic criteria for sarcopenia typically include assessments of skeletal muscle mass, muscle strength and physical performance [1, 13]. As present study is a retrospectively designed, we were only able to measure one of these criteria, namely muscle mass, using chest CT scans. The chest CT scans of inpatients were accessible and the T4MI has been widely used to measure the body skeletal muscle mass, and Moon et al. proposed the chest CT reference values for diagnosis of sarcopenia of Asian population [15]. We did not differentiate between primary and secondary sarcopenia in this cohort. The cohort consisted of individuals older than 60 years with no malignant diseases, suggesting that the loss of skeletal muscle might be age-related, indicating primary sarcopenia. This limitation might affect the comprehensive evaluation of sarcopenia in our study. Second, it’s important to note that this is a retrospective study conducted among RCT inpatients, which could introduce selective bias into our findings. To gain a more comprehensive understanding of how sarcopenia affects the occurrence of RCTs, the size of RCTs, and functional rehabilitation after repair, future research should consider investigating a larger sample of elderly individuals from diverse backgrounds in real-world settings. Third, the patient cohort in this study consisted exclusively of an Asian population. Therefore, our findings may not be representative of other racial or ethnic groups, and caution should be exercised when generalizing these results to different populations. Finally, although we found that RCT patients with sarcopenia had significantly higher levels of CRP and ESR, the underlying pathophysiological mechanisms driving this relationship remain unclear. Further research is needed to investigate these mechanisms and better understand the connections between sarcopenia, chronic inflammation, and shoulder function in RCT patients.

## Conclusions

The low skeletal muscle mass is associated with inferior ROM of the shoulder and per- and post-operative shoulder function, which means that surgeons should notice the sarcopenia condition before surgery performed and post-operative rehabilitation, especially for elderly female patients. A more personalized postoperative rehabilitation program may help improve shoulder joint function recovery in elderly RCT patients with sarcopenia. Low skeletal muscle mass does not demonstrate a significant relationship with the size of RCT.

## Data Availability

No datasets were generated or analysed during the current study.

## Abbreviations

RCT: rotator cuff tears
CT: computed tomography
CSA: cross-sectional area
ROM: range of motion
BIA: bioelectrical impedance analysis,
DXA: dual-energy X-ray absorptiometry
MRI: magnetic resonance imaging
T4CSA: the level 4 thoracic cross-sectional area
T4MI: level 4 thoracic muscle index
CRP: C-reactive protein
ESR: erythrocyte sedimentation rate
EWGSOP2: European Working Group and Sarcopenia in Older People
